# Genome-Guided Identification of Organohalide-Respiring *Deltaproteobacteria* from the Marine Environment

**DOI:** 10.1128/mBio.02471-18

**Published:** 2018-12-18

**Authors:** Jie Liu, Max M. Häggblom

**Affiliations:** a Department of Biochemistry and Microbiology, School of Environmental and Biological Sciences, Rutgers, The State University of New Jersey, New Brunswick, New Jersey, USA; Pacific Northwest National Laboratory; University of Toronto; University of Tennessee

**Keywords:** *Deltaproteobacteria*, anaerobes, marine, organohalide respiration

## Abstract

Organohalide compounds are widespread in the environment as a result of both anthropogenic activities and natural production. The marine environment, in particular, is a major reservoir of organohalides, and reductive dehalogenation is thought to be an important process in the overall cycling of these compounds. *Deltaproteobacteria* are important members of the marine microbiota with diverse metabolic capacities, and reductive dehalogenation has been observed in some *Deltaproteobacteria*. In this study, a comprehensive survey of *Deltaproteobacteria* genomes revealed that approximately 10% contain reductive dehalogenase (RDase) genes, which are found within a common gene neighborhood. The dehalogenating potential of select RDase A-containing *Deltaproteobacteria* and their gene expression were experimentally verified. Three *Deltaproteobacteria* strains isolated from marine environments representing diverse species, Halodesulfovibrio marinisediminis, Desulfuromusa kysingii, and Desulfovibrio bizertensis, were shown to reductively dehalogenate bromophenols and utilize them as terminal electron acceptors in organohalide respiration. Their debrominating activity was not inhibited by sulfate or elemental sulfur, and these species are either sulfate- or sulfur-reducing bacteria. The analysis of RDase A gene transcripts indicated significant upregulation induced by 2,6-dibromophenol. This study extends our knowledge of the phylogenetic diversity of organohalide-respiring bacteria and their functional RDase A gene diversity. The identification of reductive dehalogenase genes in diverse *Deltaproteobacteria* and confirmation of their organohalide-respiring capability suggest that *Deltaproteobacteria* play an important role in natural organohalide cycling.

## Abstract (precis)

The marine environment is a major reservoir for both anthropogenic and natural organohalides, and reductive dehalogenation is thought to be an important process in the overall cycling of these compounds. Here we demonstrate that the capacity of organohalide respiration appears to be widely distributed in members of marine *Deltaproteobacteria*. The identification of reductive dehalogenase genes in diverse *Deltaproteobacteria* and the confirmation of their dehalogenating activity through functional assays and transcript analysis in select isolates extend our knowledge of organohalide-respiring *Deltaproteobacteria* diversity. The presence of functional reductive dehalogenase genes in diverse *Deltaproteobacteria* implies that they may play an important role in organohalide respiration in the environment.

## INTRODUCTION

Organohalides are widespread in the environment as a result of both anthropogenic and natural sources. Many organohalides, for example, trichloroethene (TCE), polychlorinated biphenyls (PCBs), polybrominated diphenyl ethers (PBDEs), and brominated flame retardants (BFRs), have been widely used in industry, households, and/or agriculture and are problematic environmental pollutants (1). Over 5,000 naturally produced organohalides have also been identified, originating from diverse biogenic sources and geogenic activities (2). Microbes are now recognized to play a key role in the cycling of these organohalides through both halogenation and dehalogenation processes (1, 3, 4). Of particular interest is the process of respiratory reductive dehalogenation in which bacteria utilize organohalides as electron acceptors for energy generation. Through this process, the halogen substituent is removed and the dehalogenation products are usually more amenable to further biodegradation and are reduced in toxicity (1, 4 – 6). This process is crucial not only for the removal of organohalide pollutants from contaminated environments but also in the cycling of natural organohalides as part of a global halogen cycle (4, 7).

Organohalide-respiring bacteria (OHRBs) have been isolated from diverse environments, including organohalide-contaminated soils and sediments as well as pristine sites (8, 9). Based on their metabolic versatility, they can be classified into facultative versus obligate OHRBs (10). The growth of obligate OHRBs, including *Dehalobacter* (in the *Firmicutes*) and *Dehalococcoides* and *Dehalogenimonas* (in the *Chloroflexi*), is restricted to organohalide respiration, while facultative OHRBs, including *Desulfitobacterium* (*Firmicutes*) and various *Proteobacteria*, are more versatile in their metabolism and can utilize diverse electron acceptors other than organohalides. Although an increasing number of OHRBs have been isolated, it is apparent that their diversity and distribution are even more extensive in the environment considering that dehalogenation activities mediated by indigenous bacteria are reported in diverse environments (9, 11). Reductive dehalogenase (RDase) genes encode the key enzymes for organohalide respiration. Typically, the respiratory RDase gene operon consists of an RDase A gene encoding the catalytic unit (RDase A), an RDase B gene encoding a putative membrane-anchoring protein, and other accessory genes involved in regulation and maturation (12). The sequence of the RDase A gene (also indicated as reductive dehalogenase homologous A, *rdhA,* in many publications) commonly contains a conserved arginine translocation (Tat) signal motif (RRXFXK) and two iron-sulfur cluster binding motifs (CXXCXXCXXXCP and CXXCXXXCP motifs) (10, 13). In addition to respiratory reductive dehalogenases in anaerobes, some RDases are metabolic and not involved in energy conservation, e.g., Nitratireductor pacificus pht-3B (NprdhA) and *Comamonas* sp. 7D-2 (BhbA) (14, 15). The crystal structures of both kinds of reductive dehalogenases indicated the presence of a corrinoid cofactor in the active center (15, 16).

RDase A genes have been detected in marine sediments using specific PCR primers and metagenomics analyses (8, 17 – 22). The prevalence of RDase A genes in pristine marine environments suggests that a widespread distribution of OHRBs and organohalide respiration may be an important energy-yielding metabolic pathway for anaerobic marine bacteria, which makes it important to study OHRBs from diverse species to gain a better understanding of the role of OHRBs in the global cycling of organohalides. Deltaproteobacteria have diverse metabolisms and are ubiquitously present in the environment. Based on an earlier survey of RDase A genes in 208 Deltaproteobacteria genomes, approximately 10% of the sequenced Deltaproteobacteria contained RDase A genes in their genomes, suggesting their potential ability for organohalide respiration (23). Metagenomic analysis data also indicated that Deltaproteobacteria were dominant members of debrominating enrichment cultures derived from deep ocean sediments (19). The presence of putative RDase A genes is an indicator of potential organohalide respiration; however, the dehalogenating activity and gene functionality should be experimentally verified.

In this study, we analyzed the frequency and diversity of RDase A genes in 556 published Deltaproteobacteria genomes and examined the common features of the reductive dehalogenase gene clusters identified in their genomes. Three representative Deltaproteobacteria, not previously recognized with organohalide respiration ability, were investigated for their dehalogenating ability. Transcript analysis was also conducted to confirm the expression of RDase A in these Deltaproteobacteria in response to organobromides.

## RESULTS

### A survey of reductive dehalogenases in *Deltaproteobacteria*.

In a search of the JGI Integrated Microbial Genomes and Microbiome Samples (IMG/MER) genome database (May 2017), 556 annotated genomes were grouped in the Deltaproteobacteria. When searching for genes annotated with (potential) reductive dehalogenase function, 9% of these genomes (50 of 556) were found to contain at least one putative RDase A gene and a total of 80 putative RDase A genes were found in these different Deltaproteobacteria genomes. The similarities among these RDase A amino acid sequences are in the range of 10% to 100%. The prevalence of RDase genes in members of the Deltaproteobacteria was higher than that of other *Proteobacteria*, e.g., 7.8% in *Alphaproteobacteria*, 1.4% in *Betaproteobacteria*, 0.14% in *Epsilonproteobacteria*, and 0.4% in *Gammaproteobacteria.* In order to distinguish potential organohalide-respiring bacteria among characterized Deltaproteobacteria, 35 RDases from 19 pure culture isolates were chosen for comparison with functionally characterized RDases from non-Deltaproteobacteria (the details of their genome information are shown in Table S1 and Table S2 in the supplemental material). A tree with all 80 RDases from both isolates and metagenomics data is also shown in the supplemental material (Fig. S1).

10.1128/mBio.02471-18.1 FIG S1 Phylogenetic tree of 80 RDases from *Deltaproteobacteria* isolates and metagenomics in JGI with functionally characterized RDase As. Download FIG S1, PDF file, 0.1 MB. Copyright © 2018 Liu and Häggblom. 2018 Liu and Häggblom This content is distributed under the terms of the Creative Commons Attribution 4.0 International license.

10.1128/mBio.02471-18.4 TABLE S1 The genomic information of *Deltaproteobacteria* examined in this study and details of their reductive dehalogenase genes. Download Table S1, PDF file, 0.1 MB. Copyright © 2018 Liu and Häggblom. 2018 Liu and Häggblom This content is distributed under the terms of the Creative Commons Attribution 4.0 International license.

**FIG 1:**
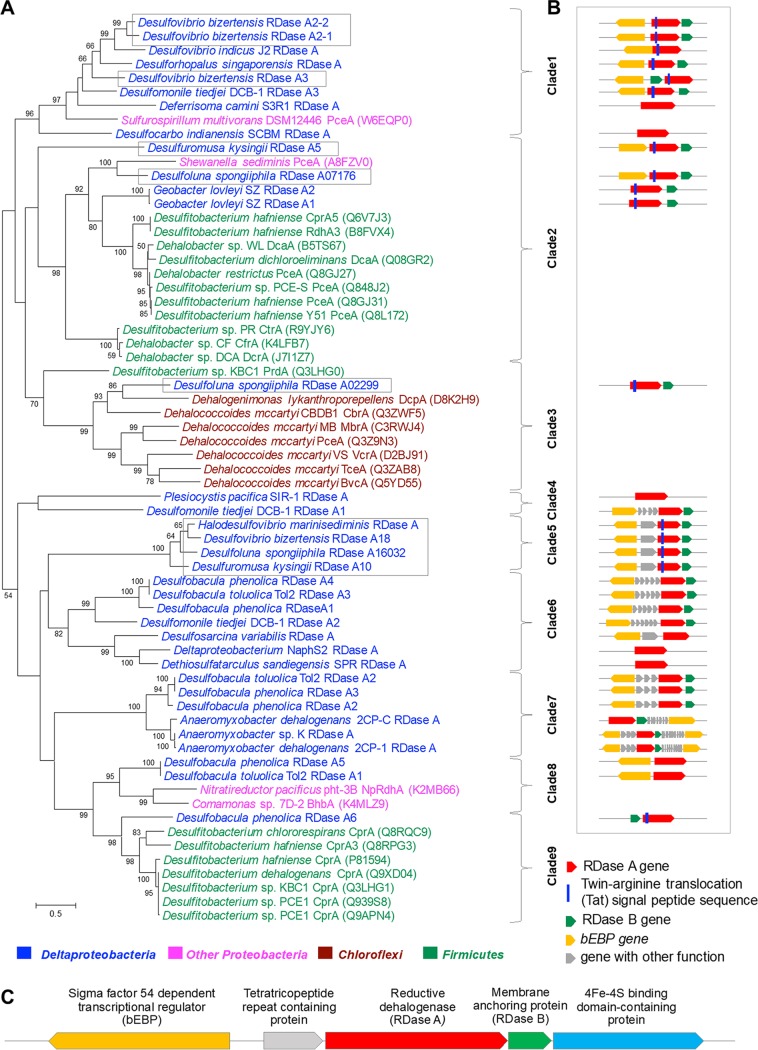
Phylogeny of putative RDase A amino acid sequences of 19 *Deltaproteobacteria* isolates and characterized reductive dehalogenases (A), the structure of *Deltaproteobacteria* reductive dehalogenase gene clusters (B), and detailed annotation of clade 5 RDase gene clusters (C). The initial tree(s) for the heuristic search was obtained by applying neighbor-joining (NJ) and advanced NJ (BioNJ) algorithms to a matrix of pairwise distances estimated using a Jones-Thornton-Taylor (JTT) model and then selecting the topology with superior log likelihood value. The maximum-likelihood tree is drawn to scale, with branch lengths measured in the number of substitutions per site. The analysis involved 65 amino acid sequences. All positions with less than 60% site coverage were eliminated, i.e., fewer than 40% alignment gaps, missing data, and ambiguous bases were allowed at any position. There were a total of 489 aa positions in the final data set. The RDase As are highlighted with different colors based on the phyla. The clades were designated based on the tree node rather than a fixed sequence cutoff. For the reductive dehalogenase gene cluster structure (B), the arrows indicate the gene functions and orientation. The sizes of those symbols are not true to real gene length. RDase A genes examined in more detail in this study and our previous study on *D. spongiiphila* are highlighted in a box.

10.1128/mBio.02471-18.5 TABLE S2 The genome information of the organohalide-respiring bacteria represented in Fig. 1. Download Table S2, PDF file, 0.1 MB. Copyright © 2018 Liu and Häggblom. 2018 Liu and Häggblom This content is distributed under the terms of the Creative Commons Attribution 4.0 International license.

According to the RDase A classification system proposed by Hug et al. (12), the Deltaproteobacteria RDase As could not be assigned to existing groups with a cutoff of 90% identity except for six RDase As (Geobacter lovleyi SZ RDase A1 and RDase A2 in ortholog group 41, Anaeromyxobacter dehalogenans 2CP-1 RDase A and *Anaeromyxobacter* sp. strain K RDase A in ortholog group 42, and deltaproteobacterium NaphS2 RDase A and Anaeromyxobacter dehalogenans 2CP-C RDase A in an unassigned group) that were already included in their most recent database (April 2018). Here, we constructed phylogenetic trees of characterized and putative Deltaproteobacteria RDase As as shown in Fig. 1A. The RDase As can be divided into nine clades based on the tree nodes rather than a fixed sequence identity cutoff. Most of the functionally characterized RDase As are from the *Firmicutes* (clades 2 and 9) and *Chloroflexi* (clade 3). RDase As from the *Chloroflexi* are grouped together in clade 3 with an identity range of 19.5 to 46.5%, while RDase As from *Firmicutes* are grouped in clade 2 (24.5 to 99.8% identity) and clade 9 (60.2 to 99.8% identity). Most of the putative RDase As from the Deltaproteobacteria grouped together with other *Proteobacteria* and were separated into several clades (clades 1, 4, 5, 6, 7 and 8). Desulfoluna spongiiphila RDase A02299 shared the highest identity with RDase As in the *Chloroflexi* clade (clade 3), while Desulfobacula phenolica RDase A6 shared an identity of 40% with the RDase As in the *Firmicutes* clade (clade 9). RDase A07176 from *D. spongiiphila* and RDase As from Geobacter lovleyi SZ grouped within *Firmicutes* clade 2.

### Reductive dehalogenase gene clusters in *Deltaproteobacteria*.

**FIG 2:**
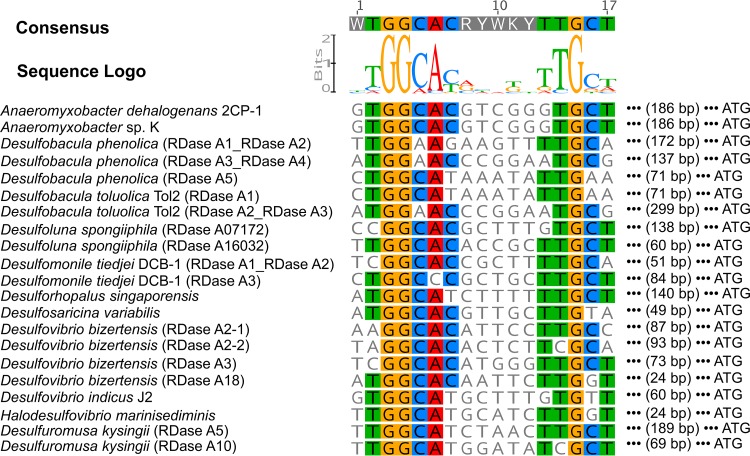
Potential sigma-factor-54 holoenzyme binding sites in the promoter region of putative reductive dehalogenases with a sigma-factor-54-dependent transcriptional activator gene nearby as shown in Fig. 1. The potential binding sites were identified based on the binding motif in the potential promoter region.

The reductive dehalogenase gene clusters of Deltaproteobacteria were explored for their common features (Fig. 1B). The conserved arginine translocation (Tat) signal was found in the N-terminal region of 16 of 35 putative RDase A genes. The lack of the Tat motif indicates that these RDase As may be cytoplasmic rather than periplasmic (24). The RDase A gene is usually associated with RDase B, which encodes a putative membrane-anchoring protein. In total, 26 of 35 Deltaproteobacteria RDase A genes have an RDase B gene in close association, indicating that the encoded reductive dehalogenase is likely a membrane-associated protein. The RDase B gene is located downstream of the RDase A gene with two exceptions, the Desulfovibrio bizertensis RDase A3 gene and the Desulfobacula phenolica RDase A6 gene, which have RDase B upstream. Genes encoding sigma factor-54 (σ^54^)-dependent transcriptional activators (also called bacterial enhancer-binding protein [bEBP]) were frequently found to be present near the Deltaproteobacteria RDase gene clusters (Fig. 1B), unlike what has been reported for *Dehalococcoides* and *Dehalobacter*. In total, 22 bEBP genes were found in 31 RDase gene clusters. These findings indicate that the transcription of these RDase gene operons in the Deltaproteobacteria may be σ^54^ dependent rather than σ^70^ dependent. In contrast to the σ^70^ holoenzyme (Eσ^70^) that recognizes and binds to conserved −10 and −35 promoter elements, Eσ^54^ binds to −12 and −24 conserved elements (YTGGCACGRNNNTTGCW), and the initiation of transcription requires the assistance of bEBPs (25). The potential Eσ^54^ binding sites were identified in these RDase gene operon promoter regions (Fig. 2), providing further evidence that the transcription of these RDase gene operons in Deltaproteobacteria may be σ^54^ dependent.

Certain RDase gene clusters were found to be conserved in some Deltaproteobacteria species. The three RDase As of Desulfobacula phenolica are not only nearly identical (>99% identity) to those of Desulfobacula toluolica but are also highly similar in gene cluster components. Moreover, the RDase gene clusters in clade 5 have similar components with high RDase A gene identity (>68%) as shown in Fig. 1C. Although from different species, all these gene clusters contain a σ^54^-dependent transcriptional regulatory gene, a tetratricopeptide-repeat-containing protein gene, and reductive dehalogenase genes. These similarities in operon components and sequence may indicate that these gene clusters have been obtained through horizontal gene transfer. The expression of genes in this clade was studied in further detail.

### Phylogeny of RDase A*-*containing *Deltaproteobacteria* isolates.

Six Deltaproteobacteria genera have previously been reported to contain members with dehalogenating ability, namely, *Anaeromyxobacter*, *Geobacter*, *Desulfomonas*, *Desulfomonile*, *Desulfoluna*, and *Desulfovibrio* (4). Of these organohalide-respiring Deltaproteobacteria, six strains have available genomes in JGI and are included in our survey. The remaining 13 RDase A-containing Deltaproteobacteria species cover 11 genera, 10 of which have never been reported to contain organohalide-respiring members (see Fig. 4 and Table S3).

10.1128/mBio.02471-18.6 TABLE S3 The 16S rRNA gene database source of OHRBs in Fig. 4 and their substrate range. Download Table S3, PDF file, 0.2 MB. Copyright © 2018 Liu and Häggblom. 2018 Liu and Häggblom This content is distributed under the terms of the Creative Commons Attribution 4.0 International license.

### Reductive dehalogenation properties of selected *Deltaproteobacteria*.

Three Deltaproteobacteria species, Desulfovibrio bizertensis, Halodesulfovibrio marinisediminis, and Desulfuromusa kysingii, were chosen for examination of their dehalogenating activity because (i) these species represent diverse genera and have been isolated from the marine environment but from different geographic locations and (ii) they all contain a complete structurally similar RDase gene cluster with RDase accessory genes but variable in sequence (clade 5). None of these species were previously reported to show dehalogenating activity (26 – 28).

The physiological properties reported in the original species descriptions, the genome information, protein yield, and their dehalogenating activity tested in this study are summarized in Table 1. *D. bizertensis* and *H. marinisediminis* are sulfate-reducing bacteria that can grow with lactate as the carbon source. *D. kysingii* is a sulfur-reducing bacterium and is phylogenetically and physiologically distinct from *D. bizertensis* and *H. marinisediminis*. Their genomes are between 3 and 4 Mbp, which is smaller than *D. spongiiphila* AA1.

**TABLE 1:** Summary of physiological and genomic properties of OHRBs tested in this study and *D. spongiiphila* strain AA1

Property	Halodesulfovibrio marinisediminis DSM 17456	Desulfuromusa kysingii DSM7343	Desulfovibrio bizertensis DSM 18034	Desulfoluna spongiiphila strain AA1
Dehalogenation activity^*a*^				
2,4,6-TBP	Yes, to 4-BP	No	Yes, to 4-BP	Yes, to phenol
2,6-DBP	Yes, to phenol	Yes, to phenol	Yes, to phenol	Yes, to phenol
2,6-DCP	No	No	No	No
Protein yield (mg/mmol e^−^)^*b*^	0.69 ± 0.25	0.80 ± 0.19	0.54 ± 0.34	NA^*c*^
Exogenous cobalamin dependency	No	Yes	No	No
Inhibition by sulfate or sulfur^*d*^	No	No	No	No
Genome size (Mbp)	3.71	3.74	3.23	6.54
GC%	44.95	46.63	52.09	57.20
No. of scaffolds in draft genome	13	27	27	52
No. of RDase A genes in genome	1	2	4	3
σ^54^-regulatory gene in vicinity	Yes	Yes for both	Yes for all	Yes for 2
Potential σ^54^ binding site	Yes	Yes for both	Yes for all	Yes for 2
Total no. of σ^54^ activators in genome	35	33	29	70
Source	Marine sediment, Tokyo Bay, Japan	Mud, Kysing Fjord south of Århus	Marine sediment, Tunisia	*Aplysina aerophoba* sponge, France
Reference for isolation	39	40	38	32

a 2,4,6-TBP for 2,4,6-tribromophenol; 2,6-DBP for 2,6-dibromophenol; 2,6-DCP for 2,6-dichlorophenol.

b Protein yield assay was conducted on lactate and 2,6-DBP. Utilized electrons were calculated from concentrations of phenol and 2-BP present in culture based on two electrons provided per bromine removed.

c NA, not available.

d Sulfur for *D. kysingii* and sulfate for others.

Bromophenolic compounds are widespread in marine environments from natural sources as well as anthropogenic input (27 – 30), and debromination has previously been demonstrated in marine and estuarine sediment microcosms (13, 31) as well as isolates (32, 33). We therefore tested the dehalogenating abilities of the three selected marine Deltaproteobacteria species using 2,4,6-tribromophenol (2,4,6-TBP), 2,6-bromophenol (2,6-DBP), and 2-bromophenol (2-BP) as electron acceptors (Table 1). The dechlorinating ability was also tested with 2,6-dichlorophenol (2,6-DCP). All three strains were able to debrominate 2,6-DBP and 2-BP to phenol, but they did not dechlorinate 2,6-DCP. This specificity to bromophenol rather than chlorophenol is similar to what was shown for *D. spongiiphila* (32, 34). *D. bizertensis* and *H. marinisediminis* are also capable of debrominating 2,4,6-TBP to 2,4-DBP and 4-bromophenol (4-BP). However, there was no further debromination of 4-BP to phenol even after extended incubation (data not shown). *D. kysingii* showed no 2,4,6-TBP-debrominating activity over 18 days. In contrast, *D. spongiiphila* strain AA1 was able to completely dehalogenate 2,4,6-TBP to 2,4-DBP, 4-BP, and phenol. The growth of *D. bizertensis*, *H. marinisediminis,* and *D. kysingii* strains could be supported by lactate with 2,6-DBP as the sole electron acceptor. Lactate alone does not support the growth of these strains, as no protein yield was detectable after 24 days of incubation. In contrast, the protein yields of three strains grown on lactate and 2,6-DBP were between 0.54 and 0.80 mg per mmol electron utilized, with 2,6-DBP debrominated with the stoichiometric accumulation of phenol (Table 1 and Fig. S2).

10.1128/mBio.02471-18.2 FIG S2 Debromination of 2,6-DBP and accumulation of phenol in *Halodesulfovibrio marinisediminis* (A), Desulfuromusa kysingii (B), and Desulfovibrio bizertensis (C) culture. Download FIG S2, PDF file, 0.2 MB. Copyright © 2018 Liu and Häggblom. 2018 Liu and Häggblom This content is distributed under the terms of the Creative Commons Attribution 4.0 International license.

We additionally examined the debrominating activity of the three strains under different growth conditions using 2,6-DBP as the electron acceptor (Table 1). The debromination rates of *D. bizertensis* and *H. marinisediminis* cultures were identical with or without exogenous cobamide (vitamin B_12_ and cyanocobalamin) (Fig. S3). However, the debrominating activity of *D. kysingii* was dependent on a supply of exogenous cobamide. Compared to the debrominating activity under cobamide-rich conditions (50 µg/liter), the debrominating activity in the absence of cobamide was minimal over 80 h, indicating that exogenous cobamide is necessary for dehalogenation by *D. kysingii*. However, these results are not necessarily consistent with the genome annotations (Table S4). Although the debromination of *D. kysingii* requires exogenous cobamide, *D. kysingii* possesses a nearly complete cobamide biosynthesis pathway, as was also the case for *D. bizertensis* and *H. marinisediminis*. However, the cobamide synthesis pathway in *D. kysingii* is apparently not fully functional.

10.1128/mBio.02471-18.3 FIG S3 Dehalogenation of 2,6-DBP by Halodesulfovibrio marinisediminis (A), Desulfuromusa kysingii (B), and Desulfovibrio bizertensis (C) in the absence or presence of cobalamin (vitamin B_12_) and sulfate/sulfur as electron acceptor. Download FIG S3, PDF file, 0.1 MB. Copyright © 2018 Liu and Häggblom. 2018 Liu and Häggblom This content is distributed under the terms of the Creative Commons Attribution 4.0 International license.

10.1128/mBio.02471-18.7 TABLE S4 Putative genes involved in cobalamin biosynthesis in the genomes of organohalide-respiring bacteria tested in this study and Desulfoluna spongiiphila strain AA1. Download Table S4, PDF file, 0.2 MB. Copyright © 2018 Liu and Häggblom. 2018 Liu and Häggblom This content is distributed under the terms of the Creative Commons Attribution 4.0 International license.

The presence of sulfate did not significantly influence the debromination rate of *H. marinisediminis* (Fig. S3A). For *D. bizertensis*, sulfate did not affect the rate of 2,6-DBP debromination, but the rate of phenol formation in the culture with sulfate was higher, indicating that the subsequent debromination of 2-BP to phenol was stimulated by sulfate (Fig. S3B). This stimulation may be attributed to the biomass increase supported by sulfate reduction over a longer incubation time. For *D. kysingii*, elemental sulfur did not affect the 2,6-DBP debromination rate when cobamide was provided in the culture (Fig. S3C). These results indicate that the debrominating activity of the tested sulfate-reducing and sulfur-reducing strains is not inhibited by the presence of other available electron acceptors.

### Expression of reductive dehalogenase genes induced by 2,6-DBP in selected *Deltaproteobacteria*.

**FIG 3:**
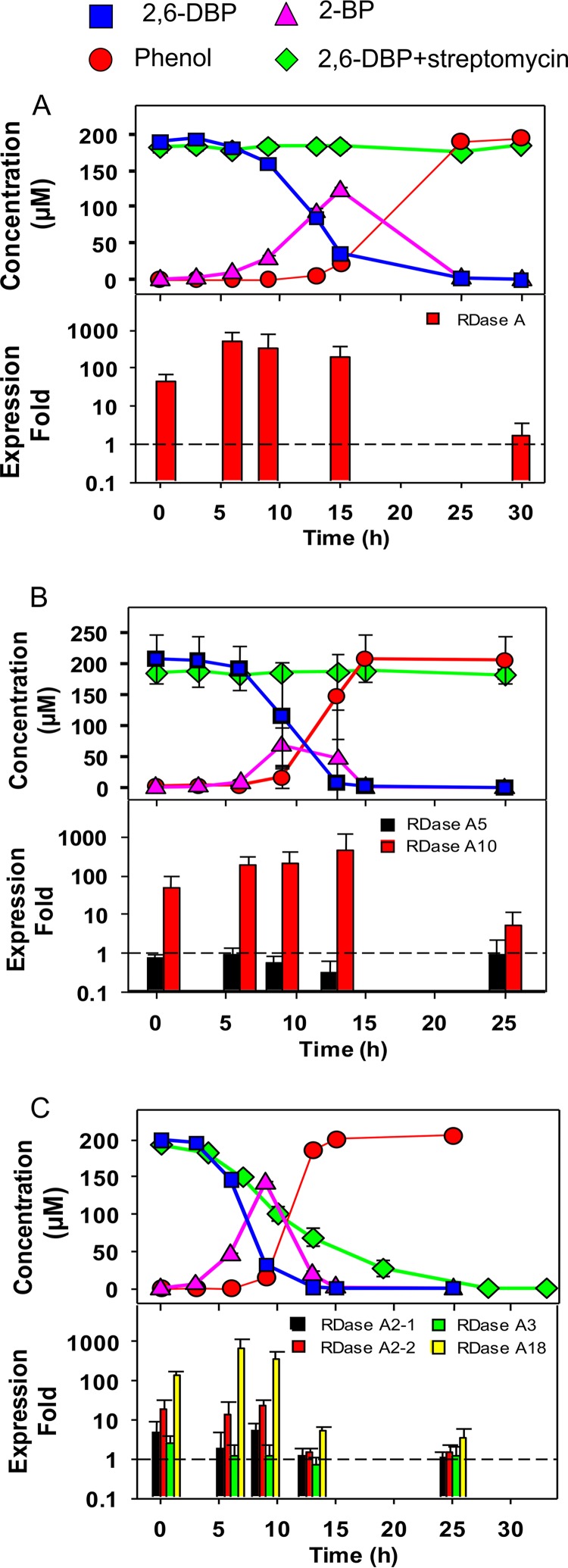
Debromination activity in 2,6-dibromophenol-induced cultures (upper panel) and the expression of RDase A genes (lower panel) of *H. marinisediminis* (A), *D. kysingii* (B), and *D. bizertensis* (C) over time. The upper panel for each strain shows the concentration of 2,6-DBP and its debromination products, 2-BP and phenol, without or with 1 mg/ml streptomycin. Error bars when larger than the symbols indicate the means and standard deviations from biological triplicates. The lower panel for each strain indicates the relative expression of RDase A genes induced by 2,6-DBP, which was normalized to the expression of the 16S rRNA gene. The *y* axis indicates the expression fold of 2,6-DBP-induced cultures compared to untreated controls. Error bars represent the standard deviations from three biological triplicates, each with RT-PCRs performed in duplicate.

To study whether the expression of the RDase A gene is inducible, streptomycin was applied to sulfate-/sulfur-grown cultures as a protein synthesis inhibitor and then spiked with 2,6-DBP (Fig. 3). The debrominating activity of *H. marinisediminis* and *D. kysingii* was almost completely inhibited by streptomycin, indicating that the expression of the RDase A gene in these two strains is downregulated during growth with sulfate or sulfur, respectively, as the terminal electron acceptor. In the *D. bizertensis* culture, 2,6-DBP was debrominated even in the presence of streptomycin but at a lower rate than the control. This indicates at least some upregulation of reductive dehalogenase enzymes when the culture was amended with 2,6-DBP.

The genome of *H. marinisediminis* contains one putative RDase A gene, whose expression was upregulated immediately after addition of 2,6-DBP (Fig. 3A). The expression level increased 50-fold in the first half hour prior to detection of debromination activity. Along with debromination activity, the expression level of this RDase A gene increased up to 400-fold and then after 30 h returned to the same level as the nonamended control.

The two RDase A genes of *D. kysingii* are located on different scaffolds of the (draft) genome and are phylogenetically distinct. The expression of the RDase A5 gene showed no difference between the control and the 2,6-DBP-amended culture, indicating that the RDase A5 gene was not induced by 2,6-DBP (Fig. 3B). In contrast, the expression of the RDase A10 gene was induced by 2,6-DBP, and the expression level increased up to 400-fold.

*D. bizertensis* has four RDase A genes in its genome, which responded differently to 2,6-DBP (Fig. 3C). The genes RDase A2-1 and RDase A2-2 are located in the close vicinity of each other in the genome and share 70% identity. The upregulation of RDase A2-1 and RDase A2-2 genes, to 5- and 20-fold, respectively, was detected from the onset of debromination and until 9 h of incubation. Then, the expression of these two RDase A genes decreased to background levels at 13 h. The expression of the RDase A18 gene was upregulated significantly (*P* < 0.05), over 600-fold, indicating that the dehalogenase encoded by the RDase A18 gene may be the major one responsible for 2,6-DBP debromination. The expression of the RDase A18 gene significantly decreased to 5-fold at 13 h, although there was still around 11 µM 2-BP remaining in the culture. The concentration of remaining 2-BP in culture may be not sufficient to maintain the expression of the RDase A18 gene at a high level. The expression of the RDase A3 gene in *D. bizertensis* was not induced by 2,6-DBP. Interestingly, the RDase A3 gene cluster has a different gene order from most other RDase gene clusters, in that its RDase B gene is located upstream of the RDase A gene. Whether this might affect the functionality of the RDase A3 gene in organohalide respiration is not known.

## DISCUSSION

**FIG 4:**
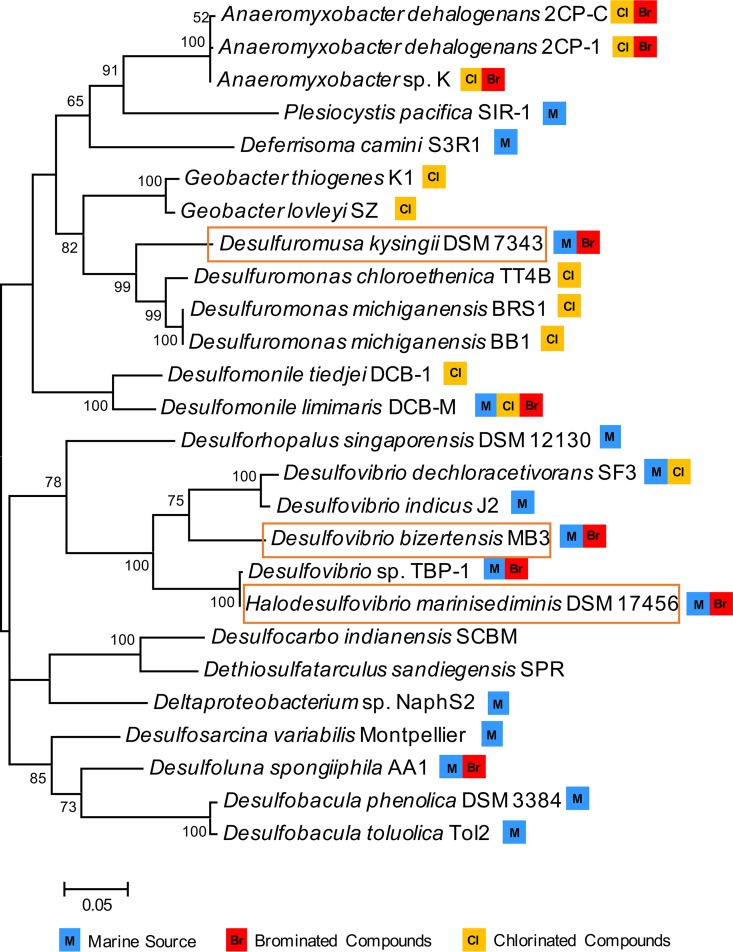
16S rRNA gene phylogenetic tree of the *Deltaproteobacteria* with dehalogenating activity or containing putative RDase A genes. The evolutionary history was inferred by using the maximum-likelihood method based on the Tamura-Nei model. Initial trees for the heuristic search were obtained by applying neighbor-joining and advanced NJ (BioNJ) algorithms to a matrix of pairwise distances estimated using a Jones-Thornton-Taylor (JTT) model and then selecting the topology with superior log likelihood value. In total, 26 sequences were used to build the tree. For organisms with multiple 16S rRNA gene copies, only one representative 16S rRNA was chosen. The detailed sequence information is listed in Table S3. All positions with less than 60% site coverage were eliminated, i.e., fewer than 40% alignment gaps, missing data, and ambiguous bases were allowed at any position. There was a total of 1,532 positions in the final data set. The marine *Deltaproteobacteria* and the ability to dehalogenate chlorinated or brominated compounds are marked with symbols as indicated. Microorganisms studied in this study are highlighted in a box.

Since the first OHRB, Desulfomonile tiedjei DCB-1, was named in 1990 (35), several others have been isolated from diverse environments (4). To date, approximately 20 organohalide-respiring Deltaproteobacteria isolates have been identified with the ability to dehalogenate chlorophenols, bromophenols, and/or chlorinated ethenes (Fig. 4). Our genomic survey of Deltaproteobacteria expanded to 556 genomes and confirms and extends the earlier analysis (23), indicating that a diverse group of Deltaproteobacteria have potential reductive dehalogenating ability encoded in their genomes. Of the RDase A-containing Deltaproteobacteria species identified in our survey, 12 of 19 are from marine environments (Fig. 4). As a major source and reservoir of natural organohalides, the marine environment appears to select for abundant and diverse organohalide-respiring microorganisms and a suite of RDase A genes (for reviews, see references 9 and 11). Although Deltaproteobacteria are frequently found in marine dehalogenating enrichment cultures, their function in the marine halogen cycle has been overlooked (15). Of the Deltaproteobacteria isolates with genomes in JGI, approximately 20% were from geographically diverse marine environments (50 of 255), and one-fourth of these marine Deltaproteobacteria contain one or more RDase A genes in their genomes (12 of 50), indicating that these are particularly prevalent in marine Deltaproteobacteria.

The application of metagenomics allows us to evaluate the dehalogenating potential in environments by screening for putative RDase A genes and eliminating the time-consuming and often difficult pure culture isolation process (17, 36). However, pure cultures of OHRBs are of significance in order to determine their physiological features and correlate with dehalogenating activity observed in environmental samples. Instead of isolating OHRBs from the environmental matrix, which is a challenge because of their low growth rates on organohalides and sensitivity to oxygen, the existing genome database can also serve as a guide for screening and identifying new OHRBs. Although RDase A genes commonly contain conserved motifs, an arginine translocation (Tat) signal motif (RRXFXK) and two iron-sulfur cluster binding motifs (CXXCXXCXXXCP and CXXCXXXCP motifs) (10, 37), their sequence identity can be lower than 10% due to the sequence diversity. The annotation accuracy of the putative RDase A genes with low identity to other known RDase A genes can be questionable and misleading for predicting RDase A gene functionality. In this case, the RDase gene cluster composition and the presence of accessory genes such as an RDase B gene and potential regulatory genes should be used as additional indicators to predict the dehalogenating functionality of the bacteria.

Three Deltaproteobacteria were selected as promising OHRBs for more detailed study because they contain complete RDase gene clusters similar to the known functional *D. spongiiphila* RDase 16032 (34). These three Deltaproteobacteria were not originally isolated for their dehalogenating activity (38, 39). *H. marinisediminis* shares high phylogenetic similarity to *Desulfovibrio* sp. strain TBP-1, which is a bromophenol-respiring bacterium isolated from estuarine sediment (33). *H. marinisediminis* was first reported to have no debrominating activity on 2,4,6-TBP (39), which is contradictory to our results. The negative result from the original report may be due to different growth or medium conditions. *D. kysingii* is an elemental sulfur-reducing bacterium isolated from marine sediment (40) but has not been previously reported to have dehalogenating ability. However, in a study of 2,4,6-TBP and 2,4,6-triiodophenol (2,4,6-TIP) dehalogenating sediments, bacteria belonging to *Desulfuromusa* were enriched and became dominant members of the bacterial community compared to control sediments (19), suggesting their role in dehalogenation. Although the Deltaproteobacteria tested in this study are from different geographic locations, their common RDase gene cluster organization and similar expression profiles during debromination of 2,6-DBP indicate that this RDase gene cluster appears to be conserved in some Deltaproteobacteria and may be important for their metabolism.

The majority of RDase As identified in the Deltaproteobacteria are distinct from previously functionally characterized RDase As, which are mainly dechlorinases from the genera *Desulfitobacterium*, *Dehalococcoides,* and *Dehalobacter* (Fig. 1). Also, most organohalide-respiring Deltaproteobacteria were previously studied for their dechlorinating abilities (Fig. 4). Brominated compounds, however, are widespread, especially in the marine environment (2). *Anaeromyxobacter* strains and Desulfomonile limimaris DCB-M were found to dehalogenate brominated aromatics (41, 42). *D. spongiiphila* strain AA1 and *Desulfovibrio* sp. TBP-1 are able to dehalogenate a variety of brominated compounds rather than chlorinated compounds (32, 33). The three marine Deltaproteobacteria tested here are functional for brominated rather than chlorinated phenols.

The analysis of the RDase gene clusters in Deltaproteobacteria genomes revealed features different from those in *Dehalococcoides* and *Desulfitobacterium* species. The regulatory mechanisms of reductive dehalogenation have been reported for *Dehalobacter*, *Desulfitobacterium,* and *Dehalococcoides* strains. The *rdhK* genes encoding CRP/FNR transcriptional regulators are commonly present in *Desulfitobacterium* and *Dehalobacter* RDase gene clusters and appear to function as transcriptional activators (43 – 46). In contrast, a *marR* regulatory gene in Dehalococcoides mccartyi is found to be associated with RDase gene clusters and acts as a negative regulator (47, 48). Except for *D. bizertensis* RDase3 with a *marR* gene, neither *CRP/FNR* nor *marR* genes were found in the RDase gene clusters of Deltaproteobacteria. Our previous analysis of *D. spongiiphila* revealed the presence of σ^54^-dependent activator (bEBP) near the two 2,6-DBP-induced RDase gene cluster (34). Similar bEBPs were also found close to the RDase A gene either directly next to or in the vicinity of the RDase As in most of the Deltaproteobacteria RDase gene clusters. The prevalence of bEBPs near the RDase gene promoter region in Deltaproteobacteria suggests that Deltaproteobacteria RDase gene operons may be regulated by a mechanism different from the *marR* or CPR/FNR systems. Sigma-factor-54-initiated transcription is widespread in bacteria (60% of bacterial genomes) to coordinate many metabolic processes (49). Anaerobic Deltaproteobacteria isolated from soil and aquatic habitats contain the highest relative number of bEBPs (normalized by genome size) among all sequenced bacterial species (50). In the genomes of RDase A-containing Deltaproteobacteria, the number of bEBPs is in the range of 27 to 88, indicating the prevalence of σ^54^-mediated transcription (see Table S1 in the supplemental material). There are few to no bEBPs in the genomes of dehalogenating *Dehalococcoides* and *Dehalobacter* spp., while none of the identified bEBPs in *Desulfitobacterium* spp. were close to the RDase gene clusters, suggesting that bEBP-associated RDase genes are unique to the Deltaproteobacteria. Whether the RDase gene operon transcription is σ^54^ initiated and whether organohalides are substrates for these bEBPs in modulating the expression warrant further investigation.

Most characterized reductive dehalogenases contain a corrinoid cofactor, which is essential for reductive dehalogenation (4, 51). The crystal structure of the RDase from Sulfurospirillum multivorans and Nitratireductor pacificus pht-3B reveals the involvement of a corrinoid cofactor in the active center (15, 16). OHRBs that are not capable of *de novo* cobamide synthesis need to obtain the cobamide through exogenous sources (52, 53). For example, *Dehalococcoides* strains which are not able to biosynthesize cobamide *de novo* were found to utilize the cobamide produced by other species grown in coculture, such as Geobacter lovleyi, which is also an organohalide-respiring member of the Deltaproteobacteria (54 – 56). Two of our tested Deltaproteobacteria strains do not require exogenous cobamide for debrominating activity, indicating that they may be able to synthesize cobamide *de novo*. Although most of the needed cobamide biosynthesis genes were found in the genomes of the tested dehalogenating Deltaproteobacteria, it does not necessarily indicate a functional cobamide biosynthesis pathway. A truncation of even a single gene involved in cobamide biosynthesis could result in a loss of cobamide biosynthesis ability (53). Similarly, the genome of *D. kysingii* contains a near-complete cobamide biosynthesis pathway; however, its debrominating activity is dependent on exogenous cobamide supplementation.

Sulfate is abundant in marine and estuarine environments, which can be utilized as an electron acceptor with production of sulfite and hydrogen sulfide. Sulfate has been found to inhibit reductive dehalogenation in enrichment cultures due to the competition between sulfate-reducing and dehalogenating bacteria (57, 58). Sulfide produced from sulfate reduction inhibited the growth and reductive dehalogenation of Dehalococcoides mccartyi 195 (59), while sulfite and thiosulfate negatively influenced the dehalogenation of Desulfomonile tiedjei in both culture and cell extracts (60, 61). Our results indicated that the debrominating activity of the three tested marine Deltaproteobacteria was not inhibited by sulfate or sulfur. These findings are consistent with previous studies showing that the dehalogenating activities of sulfate-reducing OHRBs, *Desulfovibrio* sp. TBP-1, *Desulfomonile limimaris* DCB-M, and Desulfoluna spongiiphila AA1 of marine/estuarine origin were not inhibited by sulfate (32, 33, 41). In our survey, 11 of 12 RDase A-containing Deltaproteobacteria of marine origin are sulfate- or sulfur-reducing bacteria. Since the marine environment is a major reservoir for sulfate, sulfur, and organohalides, these OHRBs would be able to take advantage of both sulfate/sulfur reduction and respiratory dehalogenation in their natural habitats for growth.

### Conclusions.

In order to link the dehalogenating activity to functional bacterial species, it is necessary to have a better understanding of OHRB diversity. As a result of the exploration of available bacterial genomes for RDase A genes, their presence in a wide range of Deltaproteobacteria was confirmed. The marine environment is the greatest reservoir of organohalides from both anthropogenic and natural sources, and metagenomic analysis of marine sediment samples has uncovered abundant reductive dehalogenases (17, 20, 21). Three selected Deltaproteobacteria were confirmed to grow by organohalide respiration, and examination of their features extends our knowledge about OHRBs and RDase A gene diversity. Deltaproteobacteria are widespread in the environment and are involved in diverse global chemical processes, e.g., the sulfur and carbon cycles. The presence of RDase A genes in diverse Deltaproteobacteria, especially in those of marine origin, and the confirmation of their dehalogenating activity imply that they may play an important role in organohalide respiration and the cycling of organohalides in the marine environment.

## MATERIALS AND METHODS

### Survey of putative RDase A genes in *Proteobacteria* genomes.

The Joint Genome Institute Integrated Microbial Genomes and Microbiome Samples (JGI-IMG/MER) database was used to survey the occurrence of putative RDase A genes in genomes of annotated Deltaproteobacteria. Keyword “pfam13486,” which is indicative of containing the reductive dehalogenase subunit domain, was used as the “Pfam Domain Search (list)*” filter to search for RDase A genes in the database of “All Finished, Permanent Draft and Draft” Deltaproteobacteria genomes through the JGI “Find Genes” function. In total, 556 Deltaproteobacteria genomes were chosen, from which 80 putative reductive dehalogenases were found (May 2017). These putative RDase A genes hits were from 50 samples that include genomes of bacterial isolates, metagenomic sequences, and single-cell genome sequences. In a similar way, the other *Proteobacteria* classes and the *Chloroflexi* and *Firmicutes* were also surveyed for the presence of putative reductive dehalogenase genes.

The amino acid sequences encoded by RDase A genes from Deltaproteobacteria isolates together with functionally characterized RDase As whose sequences were obtained from UniProt were used to construct phylogenetic trees. The genes surrounding these RDase A genes were also examined to explore the potential regulatory genes. The amino acid sequence alignment of RDase A and the alignment of 16S rRNA gene sequences were performed using Clustal W alignment with Cost Matrix Gonnet in MEGA 7 (62).

### Identification of sigma-factor-54-related genes and binding sites.

The genomes of the selected Deltaproteobacteria were downloaded from JGI-IMG/MER with annotations. The surrounding RDase gene regions were examined for potential MarR and sigma-factor-54-dependent transcriptional regulators. For the RDase genes with a sigma-factor-54-dependent transcriptional regulator, the binding site was searched in the promoter regions for the YTGGCACGRNNNTTGC motif in Geneious. Obtained potential binding sites were further manually examined.

### Bacterial strains and growth conditions.

Desulfovibrio bizertensis DSM18034, Halodesulfovibrio marinisediminis DSM17456, and Desulfuromusa kysingii DSM7343 were obtained from the German Collection of Microorganisms and Cell Cultures (DSMZ, Braunschweig, Germany) in freeze-dried form. The cultures were revived by inoculation into anaerobic medium under oxygen-free condition and incubated at 27°C under a headspace of N_2_ until visible growth was observed. The anaerobic growth medium contained minimal salts, reductant (0.5 g/liter sodium sulfide nonahydrate), and 25 g/liter NaCl as described previously (34). For *D. bizertensis* and *H. marinisediminis*, 30 mM lactate was utilized as carbon source and electron donor with 20 mM sulfate as electron acceptor. For *D. kysingii,* 10 mM fumarate was added to support growth with 1 g/liter elemental sulfur as electron acceptor. The growth of the cultures occurred at either room temperature or 27°C. Elemental sulfur was prepared using fine, homogeneous sulfur powdered in anaerobic medium. The sulfur was not dissolved, but the slurry was thoroughly shaken before addition to the growth medium.

### Dehalogenating activity of tested strains under different conditions.

The Deltaproteobacteria strains were initially pregrown on lactate and sulfate-sulfur in anaerobic medium containing 50 µg/liter of cobamide (in the form of vitamin B_12_). In order to remove cobamide or sulfate from the cultures, 30 ml of *D. bizertensis* and *H. marinisediminis* culture pregrown on sulfate and lactate was centrifuged for 5 min at 8,000 × *g* to collect the cell pellets; these were washed twice and resuspended into cobamide-free medium. In order to reduce cobamide and sulfur in the *D. kysingii* culture to a low level, successive transfers were made into cobamide-free and sulfur-free medium. These cultures were then used to set up the experiments to test for the influence of cobamide and an additional electron acceptor (sulfate or sulfur) on dehalogenating activity.

*D. bizertensis, H. marinisediminis,* and *D. kysingii* cells were inoculated into medium containing lactate and 2,6-DBP. The consumption of 2,6-DBP and production of 2-BP and phenol were used as indicator of viability. When 2,6-DBP was depleted, another 200 µM 2,6-DBP was refed to support growth. The cultures were transferred into fresh medium after 5 refeedings of 2,6-DBP for a total of three culture transfers. To prove that the reductive dehalogenation supports the culture growth as a respiratory process, 10 ml of these stock cultures after depletion of 2,6-DBP was inoculated into 90 ml medium containing 5 mM lactate with or without 2,6-DBP as electron acceptor (370 µM). The culture with lactate and 2,6-DBP was refed twice with 2,6-DBP. After the second refeeding, 40 ml of culture was centrifuged for 15 min at 10,000 × *g* for protein analysis as described previously with modifications (33). Briefly, the collected culture was rinsed with phosphate-buffered saline (PBS; pH 7.4) to remove any medium residue. The samples were resuspended with 0.5 ml PBS and 0.5 ml 2 N NaOH and incubated at 70°C for 45 min. Then, samples were centrifuged at 13,000 × *g* for 3 min. Supernatants were neutralized with HCl and measured using the Quick Start Bradford protein assay (Bio-Rad Laboratories, Inc.). Biological culture duplicates for lactate-only controls and biological culture triplicates for lactate with 2,6-DBP were analyzed. Triplicate assays were done for each sample to determine the protein concentration.

To test for dehalogenating activity, 2 ml of washed *D. bizertensis* or *H. marinisediminis* culture was inoculated into 18 ml of cobamide-free anaerobic medium containing 2 mM lactate and about 200 μM 2,6-dibromophenol, 2,4,6-tribromophenol, or 2,6-dichlorophenol with 50 g/liter cobamide. For *D. kysingii*, 4 ml of culture was inoculated into 16 ml of cobamide-free anaerobic medium containing 2 mM fumarate and about 200 μM 2,6-DBP, 2,4,6-TBP, or 2,6-DCP with 50 g/liter cobamide.

To test the influence of cobamide and other electron acceptors, three treatments were conducted in biological triplicates for each species. The control treatment contained no additional cobamide, one treatment contained 50 µg/liter cobamide, and the third treatment contained 20 mM sulfate or 2 mM sulfur slurry together with cobamide. Samples were taken periodically and measured by HPLC to determine the concentration of organohalides and their dehalogenation products.

### Induction and expression of RDase A genes.

Cultures pregrown in 2,6-DBP-free medium were inoculated into 20 to 50 ml fresh anaerobic medium containing 1 mM lactate for *D. bizertensis* and *H. marinisediminis* as electron donor and 1 mM fumarate for *D. kysingii*. One treatment was amended with 200 μM 2,6-DBP to induce the expression of RDase A genes, while 1 mg/ml streptomycin was added to the other 2,6-DBP-amended treatment to inhibit protein synthesis. The control treatment contained an equivalent concentration of sulfate or sulfur (200 μM) as electron acceptor. In total, triplicate treatments for each species were incubated at 27°C. The cultures were sampled periodically for 2,6-DBP concentration analysis and RNA extraction.

### RNA extraction, reverse transcription, and qPCR.

Total RNA was extracted from 4 ml of culture samples using TRIzol (Ambion, Life Technologies) reagent according to the manufacturer’s instructions. The obtained RNA was treated as described previously for the downstream applications (34). The DNA-free DNA removal kit (Ambion, Life Technologies) was applied to remove gDNA contamination in RNA before reverse transcription. One microliter of RNA was used to synthesize cDNA in a 10-µl reaction mixture by using the iScript Reverse Transcription Supermix (Bio-Rad Laboratories, Inc.). The obtained cDNA was amplified using an IQ SYBR Green Supermix (Bio-Rad) in a 10-µl reaction mixture on an iCycler real-time PCR detection system (Bio-Rad). The design and specificity examination of primers used for RT-qPCR were performed in Geneious Primer 3 (see Table S5 in the supplemental material). Thermal cycling conditions for RT-qPCR were as described previously (34).

10.1128/mBio.02471-18.8 TABLE S5 Nucleotide primer sequences used for RT-PCR in this study. Download Table S5, PDF file, 0.1 MB. Copyright © 2018 Liu and Häggblom. 2018 Liu and Häggblom This content is distributed under the terms of the Creative Commons Attribution 4.0 International license.

The expression levels of the RDase A genes were calculated using a relative standard curve method. A serial dilution of gDNA of each strain was made to generate the standard curve for each gene. The transcription levels of each RDase A gene were normalized to the 16S rRNA gene. To obtain the relative expression levels, the normalized expression in 2,6-DBP-induced cultures was divided by normalized expression in control culture.

### Analytical methods.

The concentration of 2,6-DBP, 2,6-DCP, 2-BP, 2,4,6-TBP, 2,4-DBP, 4-BP, and phenol was measured by HPLC using a Sphereclone C_18_ column (250 mm by 4.6 mm; particle size, 5 μm; Phenomenex) on a Shimadzu system as described previously (34). The mobile phase consisting of methanol-water-acetic acid (70:29:1 [vol/vol/vol]) was used at an isocratic flow rate of 1 ml/min with UV absorbance detection at 280 nm.
