# Supplementary material for: Genome-Guided Identification of Organohalide-Respiring Deltaproteobacteria from the Marine Environment
Source: mBio. 2018 Dec 18;9(6):e02471-18. doi: 10.1128/mBio.02471-18 (PMC6299228; doi:10.1128/mBio.02471-18)
Supplement: FIG S1 [file mbo006184233sf1.pdf]

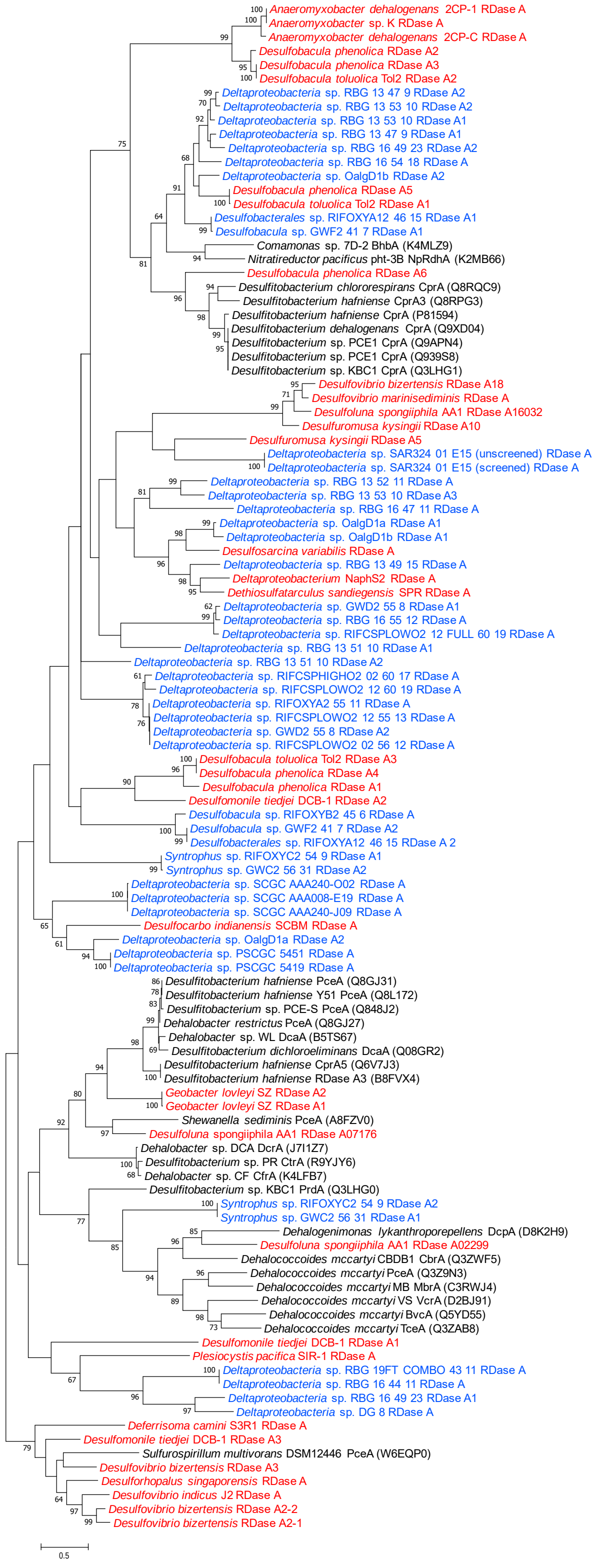

Figure S1. Phylogenetic tree of 80 RDases from D*eltaproteobacteria* isolates and metagenomics in JGI with functionally characterized RDase As. The tree construction method is the same as Figure 1. The RDases in *Deltaproteobacteria* isolates are in red, which are included in Figure 1. RDases in *Deltaproteobacteria* metagenomics data are in blue, which are not included in Figure 1.
