# Supplementary material for: Genome-Guided Identification of Organohalide-Respiring Deltaproteobacteria from the Marine Environment
Source: mBio. 2018 Dec 18;9(6):e02471-18. doi: 10.1128/mBio.02471-18 (PMC6299228; doi:10.1128/mBio.02471-18)
Supplement: TABLE S1 [file mbo006184233st1.pdf]

Table S1. The genomic information of *Deltaproteobacteria* examined in this study and details of their reductive dehalogenase genes.

Name	GOLD Analysis Project Id	NCBI Taxon ID	Source	Genome Size (Mb)	GC Content (%)	σ^{54} - Dependent Activator	RDase A gene	Gene ID	Length (bp)
Desulfovibrio bizertensis DSM18034	Ga0006634	1121442	Marine sediment, Tunisia	3.23	52.09	29	RDase A2-1	2568539639	1443
							RDase A2-2	2568539642	1440
							RDase A3	2568540098	1440
							RDase A18	2568541747	1665
Desulfovibrio indicus J2	Ga0133372	1716143	Serpentinized peridotite, Indian Ocean	3.97	63.49	27	RDase A	2688215644	1455
Desulforhopalus singaporensis DSM 12130	Ga0056957	91360	Black marine mud, Singapore	5.0	50.55	74	RDase A	2596708091	1416
Desulfoluna spongiiphila	Ga0104423	2654587882	Marine sponge	6.54	57.2	70	RDase A02299	2656130309	1449
							RDase A07176	2656131703	1287
							RDase A16032	2656133146	1680
Desulfomonile tiedjei DCB-1	Ga0025025	706587	Sludge, Wastewater, USA	6.52	50.09	71	RDase A1	2509738725	1680
							RDase A2	2509738727	1056
							RDase A3	2509739354	1416
Deferrisoma camini S3R1	Ga0026649	1125863	Deep-sea hydrothermal chimney, Pacific Ocean	4.23	70.08	29	RDase A	2517271284	1098
Geobacter lovleyi SZ	Ga0028907	398767	Freshwater sediment, South Korea	3.99	54.74	38	RDase A1	642678287	1545
							RDase A2	642678289	1545
Desulfobacula phenolica DSM 3384	Ga0066824	90732	Marine mud, Venice, Italy	4.87	41.33	55	RDase A1	2616662663	1404
							RDase A2	2616662665	1905
							RDase A3	2616662682	1920
							RDase A4	2616662684	1404
							RDase A5	2616666133	1245
							RDase A6	2616666538	1392

Table S1. Continued

Name	GOLD Analysis Project Id	NCBI Taxon ID	Source	Genome Size (Mb)	GC Content (%)	σ^{54} - Dependent Activator	RDase A gene	Gene ID	Length (bp)
Desulfobacula toluolica Tol2	Ga0012828	651182	Marine mud, USA	5.2	41.45	51	RDase A1	2524508258	1245
							RDase A2	2524509456	1920
							RDase A3	2524509458	1404
Anaeromyxobacter dehalogenans 2CP-1	Ga0027433	455488	Freshwater sediment, Michigan	5.03	74.72	38	RDase A	643593148	1923
Anaeromyxobacter dehalogenans 2CP-C	Ga0027434	290397	Tropical soil, Cameroon	5.01	74.91	40	RDase A	637861846	1923
Anaeromyxobacter sp. K	Ga0027436	447217	Soil	5.06	74.84	43	RDase A	642761794	1923
Delta-Proteobacterium NaphS2	Ga0031586	88274	Marine sediment, Germany	6.55	49.82	38	RDase A	648669933	1437
Desulfosarcina variabilis Montpellier	Ga0010238	859321	Marine mud, Montpellier, France	9.42	51.32	88	RDase A	2502438632	1440
Desulfuromusa kysingii DSM 7343	Ga0056096	37625	Marine mud, Germany	3.74	46.63	33	RDase A5	2599543621	1356
							RDase A10	2599544492	1668
Halodesulfovibrio marinisediminis DSM 1745	Ga0008187	1121457	Marine sediment	3.71	44.95	35	RDase A	2587790293	1677
Desulfocarbo indianensis SCBM	Ga0081924	1348163	Coal bed, Indiana, USA	4.91	63.06	39	RDase A	2654894503	1188
Plesiocystis pacifica SIR-1	Ga0029812	391625	Marine sandy beach, Japan	10.59	70.66	27	RDase A	641166146	1005
Dethiosulfatarculus sandiegensis SPR	Ga0077793	1429043	Methanogenic consortium, paraffin-degrading enrichment	5.93	52.06	53	RDase A	2629332177	1410
