# Supplementary material for: Genome-Guided Identification of Organohalide-Respiring Deltaproteobacteria from the Marine Environment
Source: mBio. 2018 Dec 18;9(6):e02471-18. doi: 10.1128/mBio.02471-18 (PMC6299228; doi:10.1128/mBio.02471-18)
Supplement: TABLE S2 [file mbo006184233st2.pdf]

Table S2. The genome information of the organohalide-respiring bacteria represented in Figure 1.

Name	GOLD Analysis Project Id	NCBI Taxon ID	Source	Genome Size (Mb)	GC content (%)	σ^{54} - Dependent activator	RDase A
Desulfitobacterium chlororespirans DSM 11544	Ga0008181	1121395	Compost pile, USA	5.61	47.29	40	2
Desulfitobacterium dehalogenans JW/IU-DC1	Ga0024867	756499	Freshwater pond sediment, Georgia USA	4.32	44.97	21	6
Desulfitobacterium dichloroeliminans LMG P-21439	Ga0024953	871963	Soil polluted with 1,2-DCA	3.62	44.22	12	1
Desulfitobacterium hafniense DCB-2	Ga0028500	272564	Municipal sludge, Denmark	5.28	47.54	34	7
Desulfitobacterium hafniense Y51	Ga0028501	138119	Soil contaminated with tetrachloroethene, Japan	5.73	47.36	38	1
Dehalococcoides mccartyi BAV1	Ga0028484	216389	Fresh water, Michigan	1.34	47.17	0	12
Dehalococcoides mccartyi CBDB1	Ga0028485	255470	Anoxic river sediment	1.4	47.03	0	31
Dehalococcoides mccartyi 195	Ga0028483	243164	Anaerobic sewage digestor	1.47	48.85	0	18
Dehalobacter restrictus DSM 9455	Ga0025056	871738	PCE-dechlorinating packed-bed column, Netherlands	2.94	44.56	3	24
Dehalobacter sp. CF	Ga0026847	1131462	1,1,1-TCA and TCE contaminated site	3.09	44.31	1	17
Nitratireductor pacificus pht-3B	Ga0012014	391937	Sediment, Indian Ocean	4.47	65.51	5	3
Sulfurospirillum multivorans DSM 12446	Ga0023093	1150621	Activated sludge, Germany	3.18	40.9	4	2
Dehalogenimonas lykanthroporepellens BL-DC-9	Ga0028488	552811	Waste recovery well at the Petro-Processors of Louisiana, Inc. Superfund Site	1.69	55.04	0	22
Shewanella sediminis HAW-EB3	Ga0030285	425104	Sediment at depth of 215m from an unexploded-ordinance-dumping site at Halifax	5.52	46.08	18	5
