# Supplementary material for: Genome-Guided Identification of Organohalide-Respiring Deltaproteobacteria from the Marine Environment
Source: mBio. 2018 Dec 18;9(6):e02471-18. doi: 10.1128/mBio.02471-18 (PMC6299228; doi:10.1128/mBio.02471-18)
Supplement: TABLE S3 [file mbo006184233st3.pdf]

Table S3. The 16S rRNA gene database source of OHRBs in Figure 4 and their substrate range.

Name	Substrate Range	ID	Reference
Anaeromyxobacter dehalogenans 2CP-1<br>*Anaeromyxobacter dehalogenans* 2CP-C<br>*Anaeromyxobacter* sp. K	2,6-Dichorophenol; 2,5-Dichlorophenol;<br>2-Chlorophenol; 2-Bromophenol	643594130<br>640712235<br>642762693	Sanford et al., 2002
Deferrisoma camini S3R1	Not Studied	2517270461	-
Desulfobacula phenolica DSM 3384	Not Studied	2616666760	-
Desulfobacula toluolica Tol2	Not Studied	2524508170	-
Desulfomonile tiedjei DCB-1	3-Chlorobenzoate; 2,5-Dichlorobenzoate	2509740724	DeWeerd et al., 1990
Desulforhopalus singaporensis DSM 12130	Not Studied	2596710058	-
Desulfosarcina variabilis Montpellier	Not Studied	2502441539	-
Desulfovibrio indicus J2	Not Studied	2688214528	-
Dethiosulfatarculus sandiegensis SPR	Not Studied	2629331604	-
Geobacter lovleyi SZ	Tetrachloroethene; Trichloroethene	642676148	Sung et al., 2006;<br>Wagner et al., 2012
Plesiocystis pacifica SIR-1	Not Studied	641164571	-
Deltaproteobacterium sp. NaphS2	Not Studied	AJ132804.1	-
Desulfocarbo indianensis SCBM	Not Studied	NR_126285.1	-
Desulfoluna spongiiphila AA1	2,6-Dibromophenol; 2-Bromophneol; 4-Bromophenol;<br>2,4-Dibromophenol; 2,4,6-Tribromophenol; 2-Iodophenol;<br>3-Iodophenol; 2-Bromo-4-fluorophenol; 3,5-Dibromo-4-hydroxybenzoate;<br>3,5-Dibromo-4-hydroxybenzonitrile	NR_115979.1	Ahn et al., 2009;<br>Liu et al., 2017
Desulfuromonas chloroethenica TT4B	Tetrachloroethene; Trichloroethene	NR_026012.1	Krumhlz, 1997
Desulfomonile limimaris DCB-M	3-Chlorobenzoate; 3-Bromobenzoate; 2,3-Dibromobenzoate;<br>2,5-Dibromobenzoate; 3,5-Dichlorobenzoate	NR_025079.1	Sun et al., 2001
Desulfuromonas michiganensis BB1	Tetrachloroethene; Trichloroethene	AF357915.2	Sung et al., 2003
Desulfuromonas michiganensis BRS1	Tetrachloroethene; Trichloroethene	AF357914.2	Sung et al., 2003
Desulfovibrio bizertensis MB3	2,6-Dibromophenol; 2-Bromophenol; 2,4,6-Tribromophenol;<br>2,4-Dibromophenol	NR_043808.1	This study
Halodesulfovibrio marinisediminis DSM 17456	2,6-Dibromophenol; 2-Bromophenol; 2,4,6-Tribromophenol;<br>2,4-Dibromophenol	NR_041631.1	This study
Desulfuromusa kysingii DSM 7343	2,6-Dibromophenol; 2-Bromophenol; 2,4,6-Tribromophenol;<br>2,4-Dibromophenol	2599544949	This study
Desulfovibrio sp. TBP-1	2,6-Dibromophenol; 2-Bromophenol; 2,4,6-Tribromophenol;<br>2,4-Dibromophenol; 4-Bromophenol	AF090830.1	Boyle et al., 1999
Desulfovibrio dechloracetivorans SF3	2,6-Dichlorophenol; 2-Chlorophenol	NR_025078.1	Sun et al., 2000
Geobacter thiogenes K1[a]	Trichloroacetate	NR_028775.1	De Wever et al., 2000

a-Reductive dehalogenation of Geobacter thiogenes K1 may involve a sulfur-sulfide cycle.

References:

1. Boyle AW, Phelps CD, Young LY. 1999. Isolation from estuarine sediments of a *Desulfovibrio* strain which can grow on lactate coupled to the reductive dehalogenation of 2,4,6-tribromophenol. Applied and Environmental Microbiology **65:**1133-1140.
2. De Wever H, Cole JR, Fettig MR, Hogan DA, Tiedje JM. 2000. Reductive dehalogenation of trichloroacetic acid by*Trichlorobacter thiogenes* gen. nov., sp. nov. Applied and Environmental Microbiology **66:**2297-2301.
3. Sanford RA, Cole JR, Tiedje JM. 2002. Characterization and description of *Anaeromyxobacter dehalogenans* gen. nov., sp. nov., an aryl-halorespiring facultative anaerobic *Myxobacterium*. Applied and Environmental Microbiology **68:**893-900.
4. Sun B, Cole JR, Sanford RA, Tiedje JM. 2000. Isolation and characterization of *Desulfovibrio dechloracetivorans* sp. nov., a marine dechlorinating bacterium growing by coupling the oxidation of acetate to the reductive dechlorination of 2-chlorophenol. Applied and Environmental Microbiology **66:**2408-2413.
5. Sung Y, Ritalahti KM, Sanford RA, Urbance JW, Flynn SJ, Tiedje JM, Löffler FE. 2003. Characterization of two tetrachloroethene-reducing, acetate-oxidizing anaerobic bacteria and their description as *Desulfuromonas michiganensis* sp. nov. Applied and Environmental Microbiology **69:**2964-2974.
6. DeWeerd K, Mandelco L, Tanner R, Woese C, Suflita J. 1990. *Desulfomonile tiedjei* gen. nov. and sp. nov., a novel anaerobic, dehalogenating, sulfate-reducing bacterium. Archives of Microbiology **154:**23-30.
7. Wagner DD, Hug LA, Hatt JK, Spitzmiller MR, Padilla-Crespo E, Ritalahti KM, Edwards EA, Konstantinidis KT, Löffler FE. 2012. Genomic determinants of organohalide-respiration in *Geobacter lovleyi*, an unusual member of the *Geobacteraceae*. BMC Genomics **13:**200.
8. Liu J, Lopez N, Ahn Y, Goldberg T, Bromberg Y, Kerkhof LJ, Haggblom MM. 2017. Novel reductive dehalogenases from the marine sponge associated bacterium *Desulfoluna spongiiphila.* Environmental Microbiology Reports **9:**537-549.
9. Ahn Y-B, Kerkhof LJ, Häggblom MM. 2009. *Desulfoluna spongiiphila* sp. nov., a dehalogenating bacterium in the *Desulfobacteraceae* from the marine sponge A*plysina aerophoba*. International Journal of Systematic and Evolutionary Microbiology **59:**2133-2139.
10. Krumholz LR. 1997. *Desulfuromonas chloroethenica* sp. nov. Uses Tetrachloroethylene and Trichloroethylene as Electron Acceptors. International Journal of Systematic and Evolutionary Microbiology **47:**1262-1263.
11. Sun B, Cole JR, Tiedje JM. 2001. *Desulfomonile limimaris* sp. nov., an anaerobic dehalogenating bacterium from marine sediments. International Journal of Systematic and Evolutionary Microbiology **51:**365-371.
