# Supplementary material for: Genome-Guided Identification of Organohalide-Respiring Deltaproteobacteria from the Marine Environment
Source: mBio. 2018 Dec 18;9(6):e02471-18. doi: 10.1128/mBio.02471-18 (PMC6299228; doi:10.1128/mBio.02471-18)
Supplement: FIG S2 [file mbo006184233sf2.pdf]

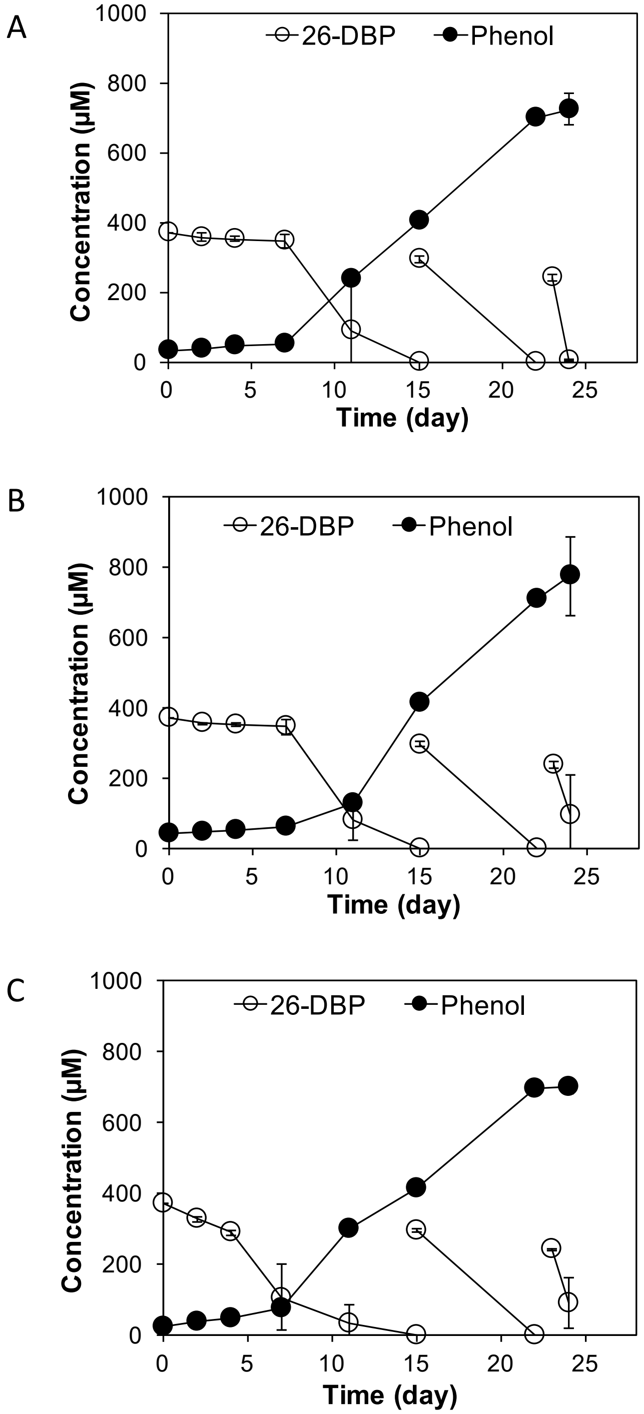

Figure S2. Debromination of 2,6-DBP and accumulation of phenol in *Halodesulfovibrio marinisediminis* (A), *Desulfuromusa kysingii* (B) and *Desulfovibrio bizertensis* (C) culture. The cultures were collected for an end point protein assay.
