# Supplementary material for: Genome-Guided Identification of Organohalide-Respiring Deltaproteobacteria from the Marine Environment
Source: mBio. 2018 Dec 18;9(6):e02471-18. doi: 10.1128/mBio.02471-18 (PMC6299228; doi:10.1128/mBio.02471-18)
Supplement: FIG S3 [file mbo006184233sf3.pdf]

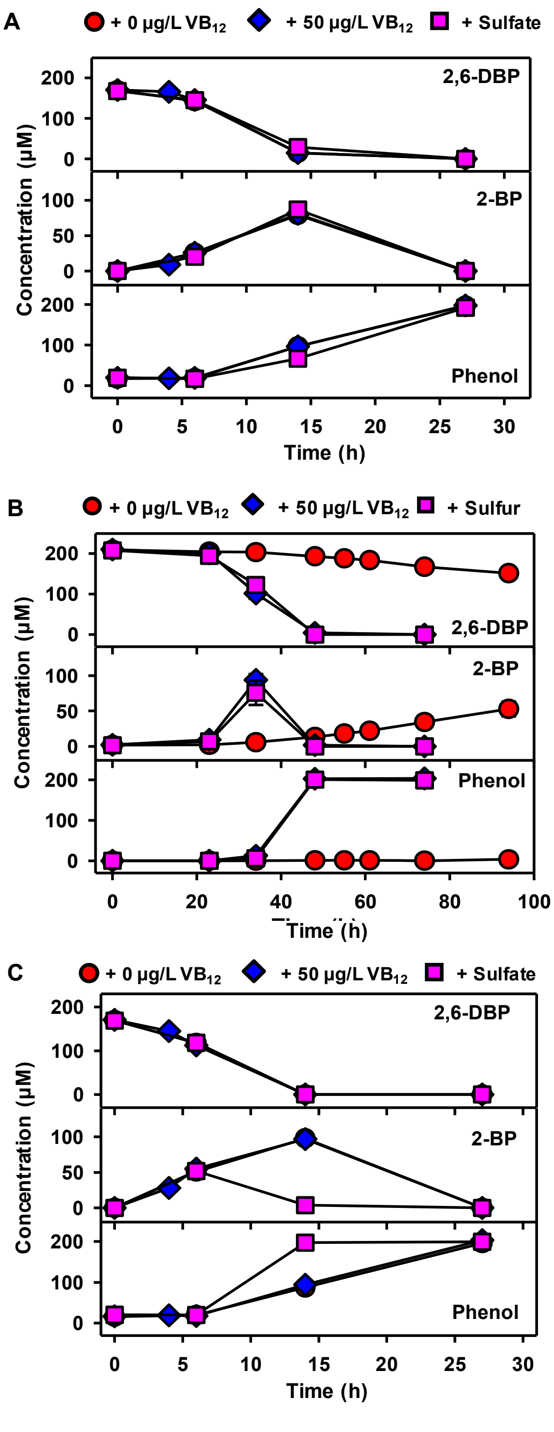

Figure S3. Dehalogenation of 2,6-DBP by *Halodesulfovibrio marinisediminis* (A), *Desulfuromusa kysingii* (B) and *Desulfovibrio bizertensis* (C) in the absence or presence of cobalamin (vitamin B_{12}) and sulfate/sulfur as electron acceptor. For each figure, the upper panel indicates the concentration of 2,6-DBP in each treatment; the middle panel indicates the concentration of 2-BP and lower panel indicates the concentration of phenol. Data points indicate the means and standard deviations of triplicate cultures.
