# Supplementary material for: Genome-Guided Identification of Organohalide-Respiring Deltaproteobacteria from the Marine Environment
Source: mBio. 2018 Dec 18;9(6):e02471-18. doi: 10.1128/mBio.02471-18 (PMC6299228; doi:10.1128/mBio.02471-18)
Supplement: TABLE S4 [file mbo006184233st4.pdf]

Table S4. Putative genes involved in cobalamin biosynthesis in the genomes of organohalide-respiring bacteria tested in this study and *Desulfoluna spongiiphila* strain AA1

Gene Abbrev.	Functional Role	***H. marinisediminis***	***Dur. kysingii***	***Dvi. bizertensis***	***Dlu. spongiiphila***
		**Gene ID**	**Gene ID**	**Gene ID**	**Gene ID**
CbiK	Sirohydrochlorin cobaltochelatase	2587791438<br>2587789883	2599543416	2568540274<br>2568540624	2656131771<br>2656134271
CbiL	Cobalt-precorrin-2 C20-methyltransferase	2587791434	2599543414<br>2599543411	2568540270	2656134277
CbiH	Cobalt-precorrin-3b C17-methyltransferase	2587790554	2599543408	2568541449	2656134283
CbiG	cobalt-precorrin 5a hydrolase	2587790553	2599543409	2568541450	2656134282
CbiF	Cobalt-precorrin-4 C11-methyltransferase	2587791567	2599543410	2568541455	2656134281
CbiD	Cobalt-precorrin-6 synthase	2587791569	2599543413	2568540852	2656134279
CbiJ	Cobalt-precorrin-6x reductase	-	2599543407	-	-
CbiET	Cobalt-precorrin-6y C5-methyltransferase	2587791568	2599543412<br>2599544127	2568540853	2656134280
CbiC	Cobalt-precorrin-8x methylmutase	2587790573	2599543417	2568538875	2656134276
CbiA	Cobyrinic acid a,c-diamide synthase	2587790574<br>2587790682	2599543418	2568540851<br>2568541122	2656134105<br>2656134275
CobA	Uroporphyrinogen-III methyltransferase	2587791030	2599543419<br>2599542566	2568541128	2656130579
CbiP	Cobyric acid synthase	2587792387	2599542341	2568541166	2656134285
CobD	L-threonine 3-O-phosphate decarboxylase	2587792472	-	-	2656134288
CbiB	Adenosylcobinamide-phosphate synthase	2587789560	2599542342	2568541021	2656134290
CobU	Adenosylcobinamide-phosphate guanylyltransferase	2587790455	2599542338	2568541742	2656134286
CobC/CblY	Alpha-ribazole-5'-phosphate phosphatase	2587791323	2599544128	2568539480	2656131162
CobT	Nicotinate-nucleotide-dimethylbenzimidazole phosphoribosyltransferase	2587790664	2599542339	2568541109	2656129606
CobS	Cobalamin synthase	2587791095<br>2587792173	2599542340	2568540769<br>2568540067<br>2568539740	2656134287
–	Glutamyl-tRNA synthetase	2587792331<br>2587790117	2599544587	2568538920<br>2568540059	2656132446<br>2656133606
HemA	Glutamyl-tRNA reductase	2587790085	2599542568	2568540320	2656134349
HemB	Porphobilinogen synthase	2587791660	2599542565	2568539875	2656134423
HemC	Porphobilinogen deaminase	2587792401	2599542567	2568541682	2656130578
HemE	Uroporphyrinogen decarboxylase	-	2599542026	2568539509	2656134671<br>2656131981<br>2656129945<br>2656133015
HemL	Glutamate-1-semialdehyde aminotransferase	2587790552	2599544468	2568541451	2656131384<br>2656132659<br>2656133626
HemN	Oxygen-independent coproporphyrinogen-3 oxidase	2587790636	2599541825<br>2599544729	2568539183	2656130302<br>2656132734<br>2656132719
HemY	Protoporphyrinogen oxidase	-	2599542035	-	-
CbiM,N,O.Q	Cobalt/nickel transport system protein	2587791554<br>2587792083<br>2587792081<br>2587792082	2599541849	2568539455<br>2568540623<br>2568540622<br>2568541056	2656132238<br>2656132234<br>2656130427<br>2656132235<br>2656132237
BtuBCDF	Vitamin B12/Fe3+ ABC transporter, permease component	2587791435<br>2587791436<br>2587791437	2599542344<br>2599542345<br>2599542346<br>2599542347	2568539097<br>2568539554<br>2568540272<br>2568539555<br>2568539098<br>2568540273<br>2568539556<br>2568539099<br>2568540271	2656131763<br>2656134273<br>2656131826<br>2656131825<br>2656131764<br>2656134272<br>2656131765<br>2656131828<br>2656134274
BtuR	Cob(I)alamin adenosyltransferase	2587789955	2599542343	-	2656131122
CysG	Siroheme synthase / Precorrin-2 oxidase	2587790087	-	2568540318	2656134347
