# Supplementary material for: Genome-Guided Identification of Organohalide-Respiring Deltaproteobacteria from the Marine Environment
Source: mBio. 2018 Dec 18;9(6):e02471-18. doi: 10.1128/mBio.02471-18 (PMC6299228; doi:10.1128/mBio.02471-18)
Supplement: TABLE S5 [file mbo006184233st5.pdf]

Table S5. Nucleotide primer sequences used for RT-PCR in this study.

Strain	Primer	Sequence (5'-3')	Target Gene	Product Length (bp)
***Halodesulfovibrio marinisediminis* DSM 17456**	653F	GAGGGAAGTGGAATTTCTGGTGTAG	16S rRNA	94
	747R	CAGTGTCAGTAATAGTCCAGGAAGT		
	725F	AGCCTATGGAGTTCAAATCACCTAA	RDase A gene	99
	824R	GGGTCAAATTTAGCAATACCAACCA		
***Desulfuromusa kysingii* DSM 7343**	887F	CGCAAGGCTAAAACTCAAAGGAA	16S rRNA	88
	975R	GGTAAGGTTCTTCGCGTTGC		
	650F	TCGTTGATAATGGGCGCGATA	RDase A5 gene	98
	748R	CGACATTTGTTTTCAGCCCGT		
	621F	TTCCACCATTCCAGAAGATCCAG	RDase A10 gene	70
	691R	CCCATTTTTCTGGAGGTTGTTCC		
***Desulfovibrio bizertensis* DSM 18034**	209F	CATGCTTTCACTTCTAGATGAGTCC	16S rRNA	91
	300R	CCTCTTAGACCAGTTATCCATCGTC		
	993F	GATTCTGACCAACATGCCGC	RDase A2-1 gene	90
	1083R	CTCGCGGGCACACTTTTTAC		
	367F	CTGTATGGCATGGAACGGATGAA	RDase A2-2 gene	84
	451R	CATCATTCCAGCTCAAAGGTCCC		
	213F	CTACCAGCGTTTTTCTACAGCAAAT	RDase A3 gene	96
	309R	CTTGATTAAATTTCTGCCATACGGT		
	1012F	CAATTCCTGAAAGAACTGGGCTATC	RDase A18 gene	66
	1078R	TCATGATTTCGTAGTTGTGACCG		
